# Impact of the COVID-19 vaccination on confusion around vaccination in general: A longitudinal study on a university population over 18 months

**DOI:** 10.1371/journal.pgph.0004066

**Published:** 2024-12-19

**Authors:** Marine Paridans, Justine Monseur, Nicolas Gillain, Eddy Husson, Gilles Darcis, Claude Saegerman, Laurent Gillet, Fabrice Bureau, Anne-Françoise Donneau, Michèle Guillaume, Benoit Pétré

**Affiliations:** 1 Research Unit Public Health: From Biostatistics to Health Promotion, University of Liège, Liège, Belgium; 2 Infectious Diseases Department, Liège University Hospital, Liège, Belgium; 3 Fundamental and Applied Research for Animal and Health (FARAH) Center, Liège University, Liège, Belgium; 4 Laboratory of Immunology-Vaccinology, FARAH, Liège University, Liège, Belgium; 5 Laboratory of Cellular and Molecular Immunology, GIGA Institute, Liège University, Liège, Belgium; PLOS: Public Library of Science, UNITED STATES OF AMERICA

## Abstract

Even before the COVID-19 pandemic, vaccine hesitancy was one of the main global public health threats. Unfortunately, the COVID-19 crisis and its associated risks only reinforced this hesitancy. This study aimed to identify to what extent the COVID-19 vaccination affected confusion around vaccination in general, its change and any associated factors. A questionnaire was distributed to the university population of Liège between April-June 2021 (Time 1) and July-September 2022 (Time 2). The impact of the COVID-19 vaccination on confusion around vaccination in general (score 0 to 100) was divided into three groups based on the tertiles of the study sample at different times and whether or not any change had been observed. Ordinal and multinomial regression analyses were performed to assess the relationship between the confusion and various determinants. The sample consisted of 491 participants. Time 1 vs Time 2, 41.3% vs 35.4% seemed to be less confused, 24.2% vs 28.7% were moderately confused and 34.4% vs 35.8% more confused, respectively. In terms of change, 19.4% of participants were less confused, 55.2% had not changed their opinion and 25.5% were more confused. The determinants causing confusion at both times and regarding change were self-perception, health literacy, past vaccination experiences and COVID-19 related factors (COVID-19 vaccination intention and trust in source of information at Time 1; preferred source of information, trust in vaccine producers and conspiratorial beliefs at Time 2; trust in vaccine producers for change). The results demonstrated that the COVID-19 vaccination impacted confusion around vaccination in general. Both unrelated and related COVID-19 factors, particularly regarding the progression of the pandemic, seem to have contributed to this confusion. Contributing factors require a personalised approach, evidence-based information being communicated with messages adapted to the situation and its evolution designed to allay individuals’ fears about vaccination.

## Introduction

Although there is no common consensus in the literature for defining and measuring vaccine hesitancy in a population [1–7], vaccine hesitancy can be defined as “‘delay in acceptance or refusal of vaccination despite availability of vaccination services.” [8, 9]. Vaccine hesitancy lies on a continuum, a decision-making process, between total acceptance of all vaccines (without any doubt) and total rejection of all vaccines (without any doubt) [3, 8–10]. This means that vaccine behaviours are not fixed and may even change over time. Doubts and concerns among people who are nevertheless vaccinated highlight that vaccination cannot be used to designate an intention to vaccinate. Vaccination intention and vaccination are not synonymous: vaccination intention is not an indicator of vaccination, and vice versa [5, 11]. Vaccination intention can, however, be seen as a determinant of vaccination and may increase when public health efforts are deployed to increase intention. The factors that can influence vaccine hesitancy have already been identified in the literature and can be separated into a number of groups. For example, the Strategic Advisory Group of Experts on Immunisation (SAGE) has divided these influencing factors into three categories: context-related (communication and media environment, anti- or pro-vaccination lobbies), individual- or group-related (personal, family and/or community members’ experience with vaccination, knowledge/awareness, health system and providers-trust and personal experience), and vaccine-specific (risk/benefit, introduction of a new vaccine) determinants [3, 8]. Betsch and collaborators [12] proposed a categorisation of the reasons for vaccine hesitancy into “5C” namely confidence in vaccines, complacency, constraints, calculation and collective responsibility. Vaccine hesitancy was even listed by the World Health Organization as one of the top ten threats to the overall health of the population [13] long before the COVID-19 pandemic [14]. In fact, a number of studies in the literature have examined vaccination hesitancy, intention and behaviour among individuals specifically in terms of COVID-19. These studies were carried out mainly among the adult population, and identified factors influencing vaccination intention and behaviour across different countries and COVID-19 vaccine doses, such as safety, side effects, trust, information sufficiency, efficacy, conspiracy beliefs, social influence, political roles, medical conditions, prior SARS-CoV-2 infection and previous flu vaccination [e.g. 15–28]. In addition, a number of studies have shown that people may have initial concerns but are ultimately vaccinated [29–39]. However, the COVID-19 pandemic, and more specifically the COVID-19 context, may have influenced vaccination behaviour and have subsequently been considered a determinant of vaccine hesitancy in general.

COVID-19 was a determining episode in the history of the vaccination of individuals due to its very specific context and widely publicised risks reinforcing general vaccine hesitancy. Indeed, the rapid development of these vaccines and the fear of potential side effects [40], fake news, misinformation and conspiracy theories [41], information about declining effectiveness in preventing infection and severe illness [42] and limited protection against variants [43] requiring several doses in a short interval of time may have had an impact on people’s perception of vaccination in general. In Belgium, COVID-19 primary vaccination of the general population (one or two doses) started in May 2021 with mainly two types of vaccines namely mRNA vaccines (PfizerBioNTech and Moderna in two doses), and viral vector vaccines (AstraZeneca in two doses and Johnson & Johnson in a single dose). Booster vaccination with mRNA vaccines for the general population began in December 2021 with the first booster vaccine and in September 2022 for the second booster vaccine with notably bivalent vaccines [44].

A number of studies have investigated the perception of vaccination in general during the COVID-19 pandemic using different parameters. Some of them have compared COVID-19 vaccination intention/attitudes to other vaccine-specific intention/attitudes/uptake during the COVID-19 pandemic and presented results which were considered controversial [e.g. 45–52], while others analysed the attitudes/beliefs towards vaccination in general during the COVID-19 pandemic namely confidence through vaccination in general [53] or the usefulness of vaccination to protect against infectious diseases [54].

Other studies observed changes in attitudes/beliefs towards vaccination in general during the first year of the pandemic (2019/2020-2021). These studies identified positive changes such as an increase in collective responsibility [55] and a decrease in complacency [55], mistrust of vaccine benefit [55], concerns about commercial profiteering [55, 56], constraints to vaccination [55] and an increase in the belief that vaccines protect public health and should therefore be mandatory [56], and/or negative changes such as increase in the calculation and worries about unforeseen future effects [55], uncertainty about vaccine safety and effectiveness and confusion about sources of trusted information [57].

These or other studies have highlighted the association between factors related to historical influences of vaccination, e.g. favourable vaccination attitudes [45], acceptance of at least one flu vaccination in (previous) flu seasons [45, 46, 48] or COVID-19 pandemic, e.g. vaccination intention against COVID-19 [54], having received COVID-19 booster doses, adverse effects resulting from COVID-19 vaccination [58], and intention/non-intention to get a specific vaccine or, finally, positive vaccine attitudes during the COVID-19 pandemic.

Most of these studies were cross-sectional studies, conducted before primary vaccination, a few at the time of primary vaccination [e.g. 47] or after a COVID-19 booster dose [e.g. 53, 58] and rare longitudinal studies conducted during the first year of the pandemic. To the best of our knowledge, no study to date has analysed how COVID-19 has impacted vaccine hesitancy in general and its change at different times of the pandemic, certainly not in Belgium or among a specific population. This study therefore provided the first opportunity for measuring confusion about vaccination in general and its evolution throughout the pandemic. Studying ways in which the COVID-19 vaccination could improve levels of confusion about vaccination in general, while considering factors more specific to the COVID-19 pandemic context, could help the government in the implementation of prevention strategies for future pandemics. Because of the possible changes between perceptions of vaccination and vaccination itself as mentioned above, this study should be seen as the first step towards understanding the impact of COVID-19 vaccination on vaccination behaviour in general. Further studies are, however, needed to measure its real impact on the same population.

The aim of this article was two-fold:

- To describe the impact of vaccination against COVID-19 and its impact on confusion surrounding vaccination, in particular in a university population, at different times during the COVID-19 pandemic as it changed; and
- To describe how the factors associated with the impact of vaccination against COVID-19 influenced confusion about vaccination in general, in a university population, at different times during the COVID-19 pandemic and how it changed during that time.

## Materials and methods

### Context and study design

The present research is part of a longitudinal study carried out among students and staff (administrative staff members, technical and manual staff members, scientific staff members and academic staff members) of the University of Liège (ULiège), Belgium, between April 2021 and December 2022, with the aim of studying SARS-CoV-2 infections, immune responses to SARS-CoV-2 infections and vaccines, and vaccine hesitancy (SARSSURV-ULiège study). The inclusion criteria were as follows: participants had to be between 18 and 67 years of age (67 being the legal retirement age in Belgium) and had to consent to participate by completing an online form. Any staff whose contract came to an end before 31 December 2021 and students (first year of Bachelor and diploma year) enrolled in the 2020–2021 academic year were excluded [59].

This present study was a 1.5 year observational longitudinal study, which met all STROBE requirements [60], carried out at two different times during the COVID-19 pandemic: between 01 April and 10 June 2021 (Time 1) and between 12 July and 22 September 2022 (Time 2).

### Study population

The population of ULiège comprises 5,633 staff members and 28,064 students. All members of the university who met the study criteria were sent a personal invitation to participate in the SARSSURV study, i.e. 3,576 staff members and 25,378 students. This was a voluntary sampling method.

The inclusion criteria for this present study were:

- Registered in the SARSSURV study between April and June 2021;
- Completed the first and the second questionnaire on the impact of COVID-19 on confusion about vaccination in general.

The exclusion criterion for this present study was:

- Prior vaccination against COVID-19.

Several e-mails were sent to repeat the invitation to take part in the study which minimised potential sources of non-response bias.

Between April and June 2021, 1,474 participants (981 staff and 493 students) were enrolled in the SARSSURV study and were invited to complete the questionnaires on socio-demographic characteristics, medical characteristics and vaccine hesitancy including the impact of COVID-19 vaccination on confusion about vaccination in general (Time 1); 27,480 either did not reply or refused to take part. Out of these participants, 213 (14.5%) had already been vaccinated against COVID-19 and were therefore excluded for Time 1 (because prior vaccination could have influenced their responses to vaccine hesitancy questions, particularly with regard to the impact of the COVID-19 vaccination on confusion about vaccination in general). Data were found to be missing for four staff members on the vaccine hesitancy questionnaire so these participants were excluded from taking part in the study. A total of 1257 (805 staff and 452 students) was included for Time 1.

**Fig 1:**
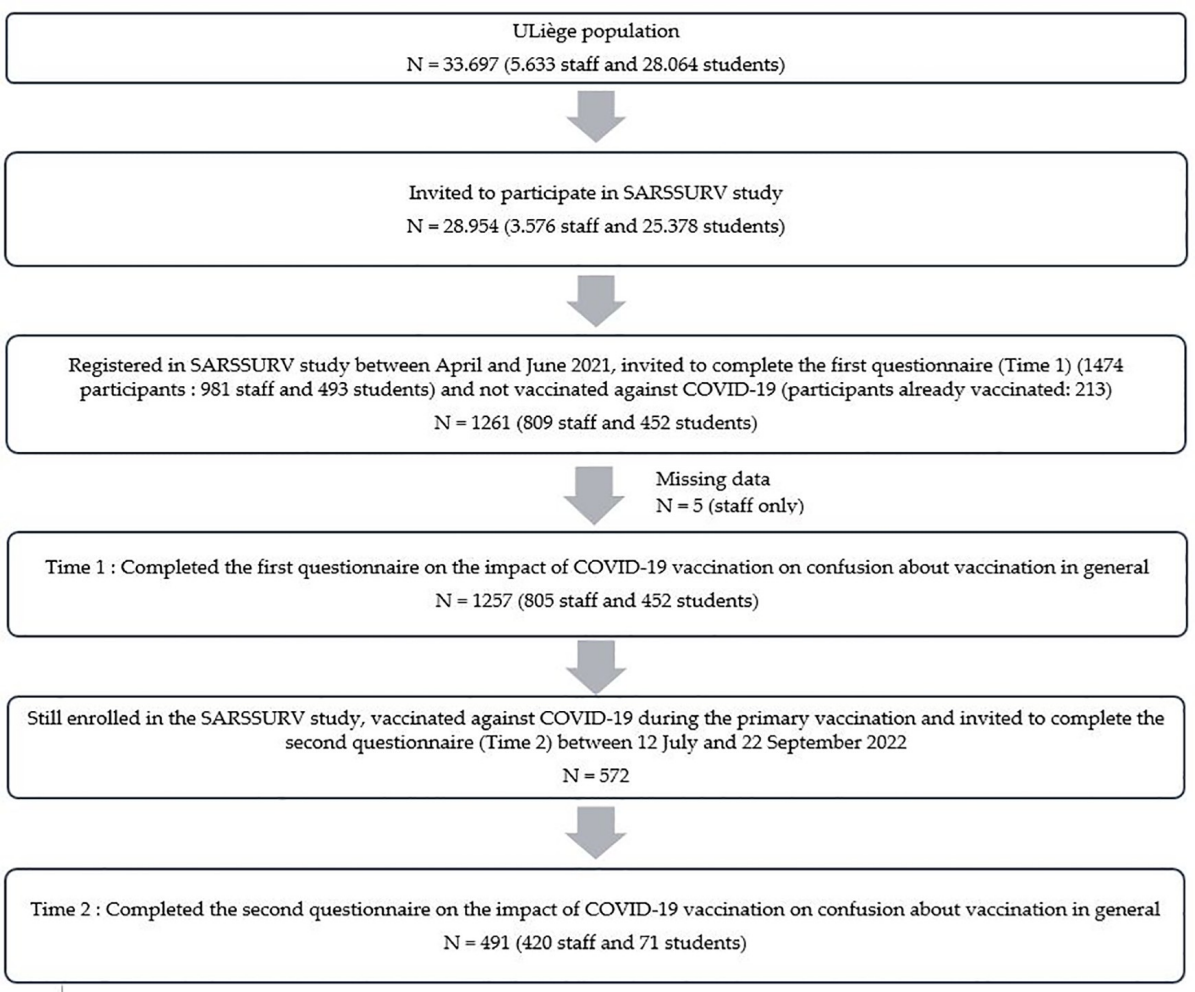
Flowchart of the study.

Based on the SARSSURV-ULiège database, participants still enrolled in the SARSSURV study and who had had their primary COVID-19 vaccination (one dose of Johnson & Johnson or two doses of AstraZeneca, Moderna, PfizerBioNTech, or Sputnik V) were invited to participate at Time 2. A total of 572 members of the University of Liège community received a personalised invitation, sent via the university’s internal mail system, to complete the questionnaire between 12 July and 22 September 2022. Finally, 491 participants (420 staff members and 71 students –85.8%) completed the questionnaire for Time 2, although a further 81 participants did not respond to the invitation or turned down the opportunity to participate (Fig 1).

In order to carry out the analyses on the same study population, only the participants who completed the questionnaire at Time 1 and Time 2 were included in this study, namely 491 participants. To recontextualise, the participants at Time 1 had not received their primary vaccination against COVID-19 and the participants at Time 2 had not previously received a second booster dose.

### Studied parameters and data collection

Data were collected from several sources at various times during the SARSSURV study. First, the sociodemographic characteristics, medical characteristics, general self-perception, health literacy, past vaccination and experience of vaccination in general, trust in authorities, vaccine producers and source of information and conspiratorial beliefs regarding COVID-19, intention to have the COVID-19 primary vaccination and SARS-CoV-2 infection and eventually primary vaccination (one or two doses). This self-reported data was collected using the self-administered questionnaires distributed via an online platform at SARSSURV enrolment, between 01 April and 10 June 2021.

Next, participants were asked to provide information, throughout the SARSSURV study, regarding any change in their vaccination status or a SARS-CoV-2 infection. They were instructed that this be reported as soon as possible and that it be done via a dedicated secure online platform or by calling/emailing a member of the research team, in order to keep the database up to date. Following any new infection or vaccination, a nurse from the research team scheduled a blood test two weeks later and then every three months. Data concerning symptoms were collected via a short questionnaire.

Intention regarding the first COVID‐19 booster vaccination was self-reported data collected using a self‐administered questionnaire that was distributed via an online platform and formed the next batch of data collected. This was carried out between 13 October and 26 December 2021.

Intention regarding the second COVID‐19 booster vaccination was self-reported data collected using a self‐administered questionnaire and was distributed via an online platform between 12 July and 22 September 2022.

Finally, information about the impact of vaccination against COVID-19 on attitudes towards vaccination in general was in the form of self-reported data collected using a self‐administered questionnaire that was distributed via an online platform at different times during the SARSSURV study: between 01 April and 10 June 2021 (Time 1) and between 12 July and 22 September 2022 (Time 2).

The questionnaires were based on a combination of models and measurement tools in the literature to determine the factors influencing vaccine hesitancy in general [8, 3, 12, 61]. They were designed and adapted to the COVID-19 episode by several members of the study group specialising in the field of research (infectious diseases and public health, etc.). The questionnaires proposed between April and June 2021 were pre-tested by ten individuals but not psychometrically validated. After the pre-test, a number of questions clarified a minor event (e.g. pain, fever) or a major event (e.g. hospitalisation).

#### Sociodemographic characteristics

The sociodemographic characteristics included institutional status (response scale: student or staff member), gender (response scale: male or female), age (response scale: open ended response in years) and highest level of education achieved (response scale: no degree, primary education, lower secondary education, upper secondary education, post‐secondary non‐tertiary education, short‐type higher education, long‐type higher education (University, and PhD).

#### Medical characteristics

The body mass index (BMI, response scale: open ended response in kg/m^2^), chronic diseases such as diabetes, hypertension, heart failure/coronary artery disease, history of stroke, liver failure/cirrhosis, kidney failure, chronic lung disease, asthma, autoimmune disease, immunodeficiency, haematologic cancer, other form of cancer, organ or cell transplant, and other health problem(s) (response scale: yes/no) were collected.

#### General self-perception

Information about the participant’s general self-perception was collected with four questions namely “Compared to other people of my age, I am a careful person (in general).”, “Compared to other people of my age, I am really careful about my health.”, “Compared to other people of my age, I am more likely to be sick.” and “Compared to other people of my age, I am an anxious person.” (response scale: Likert scale ranging from 0 (fully disagree) to 100 (fully agree)).

#### Health literacy

The single item literacy screener (SILS), slightly adapted for the purposes of this study, was used in order to assess the health literacy of ULiège staff members and students [62]. “When you read instructions, pamphlets, or other written material from your doctor or pharmacy, how often do you need help to understand the messages?” (response scale: Likert scale ranging from 0 (never) to 100 (always)).

#### Past vaccination and experience of vaccination in general

The participants were next asked about their vaccination history in general with the following two questions: “Do you have a vaccination record?” (three response modalities: yes, no and I don’t know) and “What year was your last tetanus booster?” (response scale: open ended response or I don’t know). The tetanus vaccination was chosen because it is recommended for the entire Belgian population [63], unlike the flu vaccination which is only recommended for priority populations [64]. Participants were asked these questions to obtain a general indication of interest in vaccination.

The participants were also asked about their past experience with vaccination programmes in general with the following questions “Have you ever had an adverse event after a vaccination?” (four response modalities: no, yes; a minor event (e.g. pain, fever), yes; a major event (e.g. hospitalisation), I don’t know), “Has a member of your close entourage (family, friends, colleagues) ever had an adverse event after a vaccination?” (four response modalities: no, yes; a minor event (e.g. pain, fever), yes; a major event (e.g. hospitalisation), I don’t know), “Can you think of any event(s) in the past that might discourage you from getting vaccinated in general?” (two response modalities: yes or no) and “Do you remember events in the past that persuaded you to be vaccinated?” (two response modalities: yes or no).

#### Trust in authorities, vaccine producers and source of information regarding COVID-19

Trust in authorities was collected with the question, “In your opinion, in terms of their decisions on COVID-19, what importance do the federal and regional authorities give to electoral interests, to macro-economic interests, to the physical health of the population and to the mental health of the population?” (response scale: answers ranked from most to least important).

Trust in vaccine producers was collected with the question, “I trust vaccine producers that the goals of vaccine safety and quality have not been sacrificed for the benefit of financial interests. Do you agree with this statement?” (response scale: Likert scale ranging from 0 (fully disagree) to 100 (fully agree)).

Trust in source of information was collected using the following two questions: "What is your source of information of choice in terms of the COVID-19 vaccination?" (twelve response modalities: Internet, television, printed press, entourage, social networks, political decision-makers, scientists, general practitioner, other doctors, nurses (medical centre, at home), other caregivers (physiotherapists, dentists, pharmacists) and "To what degree do you trust this source of information?" (response scale: Likert scale ranging from 0 (fully mistrust) to 100 (fully trust)).

#### Conspiratorial beliefs regarding COVID-19

The participants were asked about their conspiratorial beliefs regarding COVID-19 with the question, “Generally speaking, to what extent do you agree with the anti-vaccination arguments that are circulating?” (response scale: Likert scale ranging from 0 (fully disagree) to 100 (fully agree)).

#### Personal SARS-CoV-2 infection and vaccination history

The SARS-CoV-2 infection history included SARS-CoV-2 infections (yes/no), confirmed by a saliva‐based self‐test performed as part of the SARSSURV study [59] or by a test performed outside the study and reported by a research team participant (saliva test, nasopharyngeal test, self‐test).

The COVID‐19 vaccination history examined the participants past intention to be vaccinated against COVID‐19 using the following four questions:

- Two questions for COVID-19 primary vaccination: “On a scale of 0 to 100, what was your intention to receive the vaccination at the end of 2020?” (response scale: Likert scale ranging from 0 (no intention) to 100 (total intention)) and “On a scale of 0 to 100, what is your current intention to be vaccinated when a vaccine is offered?” (SARSSURV enrolment, April-June 2021) (response scale: Likert scale ranging from 0 (no intention) to 100 (total intention));
- One question for the first COVID-19 booster vaccination: “What is your current intention regarding accepting a booster dose when it is offered? (October-December 2021) (response scale: Likert scale ranging from 0 (no intention) to 100 (total intention))”;
- One question for a second COVID-19 booster vaccination: “On a scale of 0 to 100, what would be your intention to accept a new dose of the vaccine?” (response scale: Likert scale ranging from 0 (no intention) to 100 (total intention))” (July-September 2022).

In addition, the participants were asked to describe any symptoms they had experienced after each dose, e.g., fatigue, headache, appetite loss, muscle pain, delirium, nausea, vomiting, fever, arthralgia (joint pain), injection site pain, ipsilateral axillary lymphadenopathy (swollen lymph node(s) on the same side as the injection site), redness at the injection site, allergic reaction, and others (response scale: Likert scale ranging from 0 (no symptoms) to 10 (severe symptoms)). For this study, only symptoms declared during the last dose of the COVID-19 vaccine were considered.

The level of neutralising antibodies against COVID‐19 was measured using the most recent blood test, which was scheduled 15 days after testing positive for a SARS-CoV-2 infection or a COVID‐19 vaccination and then repeated every three months as a follow‐up. A member of the research team communicated the results to the participant via phone or letter. The neutralising antibody result was also communicated to the participants themselves in addition to its categorisation corresponding to the reference thresholds (< 1/20: Insufficient level of neutralising antibodies; ≥ 1/20 and < 1/320: Normal level of neutralising antibodies; ≥ 1/320: High level of neutralising antibodies). The current uncertainties and ongoing studies concerning the effectiveness of protection against infection by different variants, and the duration of protection over time were also shared with participants to inform them of the limited current knowledge and the need to implement the recommended preventive measures. A virus neutralisation test (VNT) was carried out with SARS-CoV-2 strain BetaCov/Belgium/SartTilman/2020/1 in 96-well plates containing Vero E6 cells (ATCC CRL-1586). Six dilutions of each heat-inactivated serum were used (1:10 to 1:320—corresponding to final testing dilutions 1:20 to 1:640). In each VNT, a positive control serum from the Belgian National Reference Centre (Sciensano) was used. Sera were mixed vol/vol with 100 TCID50/reaction of SARS-CoV-2 virus and incubated at 37°C for 1 hour. Then, the serum plus virus mixture was transferred to the cells in suspension in triplicate. The VNT relies on cytopathic effect (CPE) observation under light microscopy at day 5 post-infection. Dilutions of serum associated with CPE were considered as negative, while the absence of CPE indicated a complete neutralisation of SARS-CoV-2 inoculum (positive). Virus neutralisation titer was reported as the highest dilution of serum that neutralised CPE in 50% of the wells.

#### Impact of vaccination against COVID-19 on confusion about vaccination in general

The participants were asked about the impact of vaccination against COVID-19 on confusion about vaccination in general (response scale: Likert scale ranging from 0 (fully disagree) to 100 (fully agree)).

### Data analysis

Three dependent variables were considered for the analyses, namely the impact of COVID-19 vaccination on confusion about vaccination in general at Time 1 (April-June 2021), Time 2 (July-September 2022) and the change between Time 1 and Time 2. For Times 1 and 2, the impact of the COVID-19 vaccination on confusion about vaccination in general was categorised as lower impact, moderate impact and higher impact in line with the tertiles of the sample for Time 1. For the change, the impact of vaccination against COVID-19 on confusion about vaccination in general was either decreased (change from a higher tertile to a lower tertile), unchanged (no change in tertile) or increased (change from a lower tertile to a higher tertile).

The educational data were grouped into three categories (high school and lower, Bachelor degree, and University), and all of the SARS-CoV-2 infections were grouped into a single variable (presence or absence of infection). The number of chronic diseases and the number of symptoms experienced after the COVID‐19 vaccination were calculated. The data on adverse events after a vaccination were divided into three categories due to the low response reported in major adverse events (no event, event and I don’t know). Regarding trust in authorities, only the most importance response was considered and the data were grouped into a binary variable (electoral and macro-economic interests or health of the population). The data on source of information were grouped into four categories (medias, scientists, entourage and health professionals).

Histograms, quantile-quantile plots, and the Shapiro–Wilk tests were used to evaluate the normality of the distribution of the quantitative variables. Appropriate descriptive statistics were performed, with the frequency and percentage (%) for the qualitative variables and median (P50) and the interquartile ranges (IQR, P25–P75) reported for skewed distributed quantitative variables.

The univariate ordinal logistical regressions were performed to explore the relationship between the impact of vaccination against COVID-19 on confusion about vaccination in general and the various factors at Times 1 and 2, respectively. Regarding the change, univariate multinomial logistical regressions were used. Significant variables (p<0.05) in a univariate framework were included in the multivariate model. Due to quasi-completion of data observed in univariate cases, some variables were not considered in the multivariate analyses. The significance level was set at p < 0.05. The analyses were carried out using R statistical software version 4.1.0. The data was stored for as long as was deemed necessary in order to achieve the study’s objectives. The statistical analyses were performed on observed data only; missing data were not imputed.

### Ethical and legal aspects

The study was approved by the University Hospital of Liège Ethics Committee (reference number 2021/96, dated 26 March 2021). Written informed consent online was obtained from each participant before enrolment in the SARSSURV study. Following enrolment, a unique identification code (ID) was attributed to each participant [59]. The data were handled in a strictly confidential manner by the SARSSURV team and anonymised prior to any analysis. The compliance with data protection regulations were approved by the official University of Liège data protection officer.

## Results

### Characteristics of the study sample

As can be seen in Table 1, of the 491 participants included in the present study, 85.5% were staff members 62.7% female and 37.3% male. The median age of participants was 43.0 (33.0–51.0) years (45.0 (38.0–53.0) years for staff and 23.0 (20.5–28.0) years for students). Concerning the highest level of education, 65.0% (68.8% of staff and 42.3% of students) had a university-level education, 23.6% (26.4% of staff and 7.0% of students) had a Bachelor degree, and 11.4% (4.8% of staff and 50.7% of students) had achieved high school or lower-level education.

**Table 1:** Socio-demographic characteristics of 491 participants included in the study.

	All		Staff		Students	
Variable	N	N (%)	N	N (%)	N	N (%)
Institutional status	491					
Staff		420 (85.5)				
Student		71 (14.5)				
Gender	491		420		71	
Female		308 (62.7)		260 (61.9)		48 (67.6)
Male		183 (37.3)		160 (38.1)		23 (32.4)
Age (years)*	491	43.0 (33.0–51.0)	420	45.0 (38.0–53.0)	71	23.0 (20.5–28.0)
Highest level of education	491		420		71	
High School and lower		56 (11.4)		20 (4.8)		36 (50.7)
Bachelor degree		116 (23.6)		111 (26.4)		5 (7.0)
University		319 (65.0)		289 (68.8)		30 (42.3)
Body Mass Index (kg/m^2^)*	491	23.8 (21.5–26.7)	420	24.1 (21.7–27.2)	71	22.5 (20.5–25.1)
Chronic diseases*	491	0 (0–0)	420	0 (0–1)	71	0 (0–0)
Careful person*	491	85.0 (75.0–93.0)	420	85.0 (75.0–92.0)	71	85.0 (75.0–95.0)
Careful about health*	491	75.0 (62.0–87.0)	420	76.0 (63.8–85.3)	71	75.0 (60.0–91.5)
More likely to be sick*	491	10.0 (1.5–25.0)	420	10.0 (1.0–25.0)	71	15.0 (5.0–29.0)
Anxious person*	491	50.0 (20.0–75.0)	420	50.0 (20.0–73.3)	71	50.0 (25.0–80.0)
Health literacy*	491	8.0 (0–20.0)	420	8.0 (0–20.0)	71	8.0 (0.5–20.0)

*P50 (P25-P75)

Legend: N, number

Regarding health status, the median participant BMI was 23.8 (21.5–26.7) kg/m^2^ (24.1 (21.7–27.2) kg/m^2^ for staff and 22.5 (20.5–25.1) kg/m^2^ for students). Participants reported a median score of 0 (0–0) (0 (0–1) for staff and 0 (0–0) for students) for chronic diseases.

Regarding general self-perception, as an example, participants reported a median score of 85.0 (75.0–93.0) (85.0 (75.0–92.0) for staff and 85.0 (75.0–95.0) for students) for the statement “Compared to other people of my age, I am a careful person.” on a Likert scale ranging from 0 (fully disagree) to 100 (fully agree).

Participants reported a median health literacy score of 8.0 (0–20.0) (8.0 (0–20.0) for staff and 8.0 (0.5–20.0) for students) on a Likert scale ranging from 0 (never) to 100 (always).

Vaccination history and experience of vaccination in general, trust in authorities, vaccine producers and source of information regarding COVID-19, conspiratorial beliefs regarding COVID-19 and personal SARS-CoV-2 infection and vaccination history are presented in Table 2.

**Table 2:** Characteristics of the 491 participants included in the study.

	All		Staff		Students	
Variable	N	N (%)	N	N (%)	N	N (%)
Vaccination record	491		420		71	
Yes		278 (56.6)		221 (52.6)		57 (80.3)
No		142 (28.9)		137 (32.6)		5 (7.0)
I don’t know		71 (14.5)		62 (14.8)		9 (12.7)
Last tetanus booster	491		420		71	
I know		259 (52.8)		220 (52.4)		39 (54.9)
I don’t know		232 (47.3)		200 (47.6)		32 (45.1)
Adverse event after a vaccination	491		420		71	
Event		122 (24.9)		97 (23.1)		25 (35.2)
No event		343 (69.9)		301 (71.7)		42 (59.2)
I don’t know		26 (5.3)		22 (5.2)		4 (5.6)
Adverse event after a vaccination (entourage)	491		420		71	
Event		207 (42.2)		167 (39.8)		40 (56.3)
No event		233 (47.5)		212 (50.5)		21 (29.6)
I don’t know		51 (10.4)		41 (9.8)		10 (14.1)
Events that discouraged vaccination	491		420		71	
Yes		19 (3.9)		18 (4.3)		1 (1.4)
No		472 (96.1)		402 (95.7)		70 (98.6)
Events that encouraged vaccination	491		420		71	
Yes		112 (22.8)		94 (22.4)		18 (25.4)
No		379 (77.2)		326 (77.6)		53 (74.7)
Trust for the authorities, given their priority to	491		420		71	
Electoral-Macroeconomic interests		209 (42.6)		169 (40.2)		40 (56.3)
Physical/Mental health of the population		282 (57.4)		251 (59.8)		31 (43.7)
Trust vaccines producers*	491	60.0 (32.5–80.0)	420	60.0 (30.8–80.0)	71	60.0 (37.5–80.0)
Preferred source of information	491		419		71	
Media outlets		173 (35.3)		154 (36.8)		19 (26.8)
Scientists		210 (42.9)		172 (41.1)		38 (53.5)
Participant’s entourage		13 (2.7)		11 (2.6)		2 (2.8)
Health professionals		94 (19.2)		82 (19.6)		12 (16.9)
Trusted source of information*	490	80.0 (66.0–90.0)	419	80.0 (62.0–90.0)	71	80.0 (70.0–90.0)
Conspiratorial beliefs*	491	10.0 (0–25.0)	420	10.0 (0–25.0)	71	10.0 (0–28.5)
Prior SARS-CoV-2 infection (before Time 1)	491		420		71	
Yes		90.0 (18.3)		70 (16.7)		20 (28.2)
No		401 (81.7)		350 (83.3)		51 (71.8)
Prior SARS-CoV-2 infection (before Time 2)	491		420		71	
Yes		340 (69.3)		290 (69.1)		50 (70.4)
No		151 (30.8)		130 (31.0)		21 (29.6)
Primo-vaccination intention (end of 2020)*	491	90.0 (60.0–100)	420	90.0 (60.0–100)	71	90.0 (52.0–100)
Primo-vaccination intention (at SARSSURV enrolment)*	491	100 (82.0–100)	420	100 (83.8–100)	71	100 (80.0–100)
Booster intention*	422	100 (85.0–100)	363	100 (86.5–100)	59	99.0 (81.0–100)
Second booster intention*	491	72.0 (40.0–99.0)	420	72.5 (40.0–100)	71	71.0 (40.5–89.0)
Vaccine status (before Time 2)	491		420		71	
Complete		437 (89.0)		373 (88.8)		64 (90.1)
Incomplete		54 (11.0)		47 (11.2)		7 (9.9)
Vaccination symptoms (before Time 2)*	487	2.0 (1.0–4.0)	416	2.0 (1.0–4.0)	71	3.0 (1.0–5.0)
Neutralising antibodies (before Time 2)*	491	320.0 (160.0–1000)	420	320.0 (160.0–1000)	71	640.0 (240.0–1000)

*P50 (P25-P75)

Legend: N, number

Regarding past vaccination in general, 56.6% of participants (52.6% of staff and 80.3% of students) had a vaccination record, 28.9% (32.6% of staff and 7.0% of students) did not and 14.5% (14.8% of staff and 12.7% of students) did not know; 52.8% (52.4% of staff and 54.9% of students) knew the date of their last tetanus booster. For experience with a vaccination in general, 24.9% of participants (23.1% of staff and 35.2% of students) had personally experienced an adverse event, 69.9% (71.7% of staff and 59.2% of students) had not and 5.3% (5.2% of staff and 5.6% of students) did not know. All parameters have been summarised in Table 2.

Regarding trust in the authorities, the importance given to electoral and macroeconomic interests was seen as a priority for 42.6% of participants (40.2% of staff and 56.3% of students), as well as the physical and mental health of the population for 57.4% of participants (59.8% of staff and 43.7% of students). The median trust in vaccine producers for participants was 60.0 (32.5–80.0) (60.0 (30.8–80.0) for staff and 60.0 (37.5–80.0) for students) on a Likert scale ranging from 0 (fully disagree) to 100 (fully agree). The preferred source of information was scientists for 42.9% of participants (41.1% of staff and 53.5% of students), media outlets for 35.3% of participants (36.8% of staff and 26.8% of students), health professionals for 19.2% of participants (19.6% of staff and 16.9% of students) and their entourage for 2.7% of participants (2.6% of staff and 2.8% of students). The median for conspiratorial beliefs was 10.0 (0–25.0) for staff and 10.0 (0–28.5) for students on a Likert scale ranging from 0 (fully disagree) to 100 (fully agree).

Regarding COVID-19 personal history, 18.3% of participants (16.7% of staff and 28.2% of students) had a prior SARS-CoV-2 infection before completing the questionnaire for Time 1 and 69.3% (69.1% of staff and 70.4% of students) before completing the questionnaire for Time 2. The median past intention regarding COVID-19 vaccination was 90.0 (60.0–100) 90.0 (60.0–100) for staff and 90.0 (52.0–100) for students for primo-vaccination at the end of 2020, 100 (82.0–100) 100 (83.8–100) for staff and 100 (80.0–100) for students, for primo-vaccination at the time of enrolment in the SARSSURV study (since April 2021), 100 (85.0–100) 100 (86.5–100) for staff and 99.0 (81.0–100) for students for the booster dose (between 13 October and 26 December 2021) and 72.0 (40.0–99.0) (72.5 (40.0–100) for staff and 71.0 (40.5–89.0) for students) for the second booster dose (between 12 July and 22 September 2022). Finally, 89.0% of participants (88.8% of staff and 90.1% of students) had been fully vaccinated (primary vaccination and booster dose) before completing the second questionnaire. After the COVID-19 vaccination, participants reported a median symptoms score of 2.0 (1.0–4.0) (2.0 (1.0–4.0) for staff and 3.0 (1.0–5.0) for students). Median neutralising antibody titer was 320.0 (160.0–1000) (320.0 (160.0–1000) for staff and 640.0 (240.0–1000) for students) before Time 2.

### Impact of COVID-19 vaccination on confusion about vaccination in general and its change

**Fig 2:**
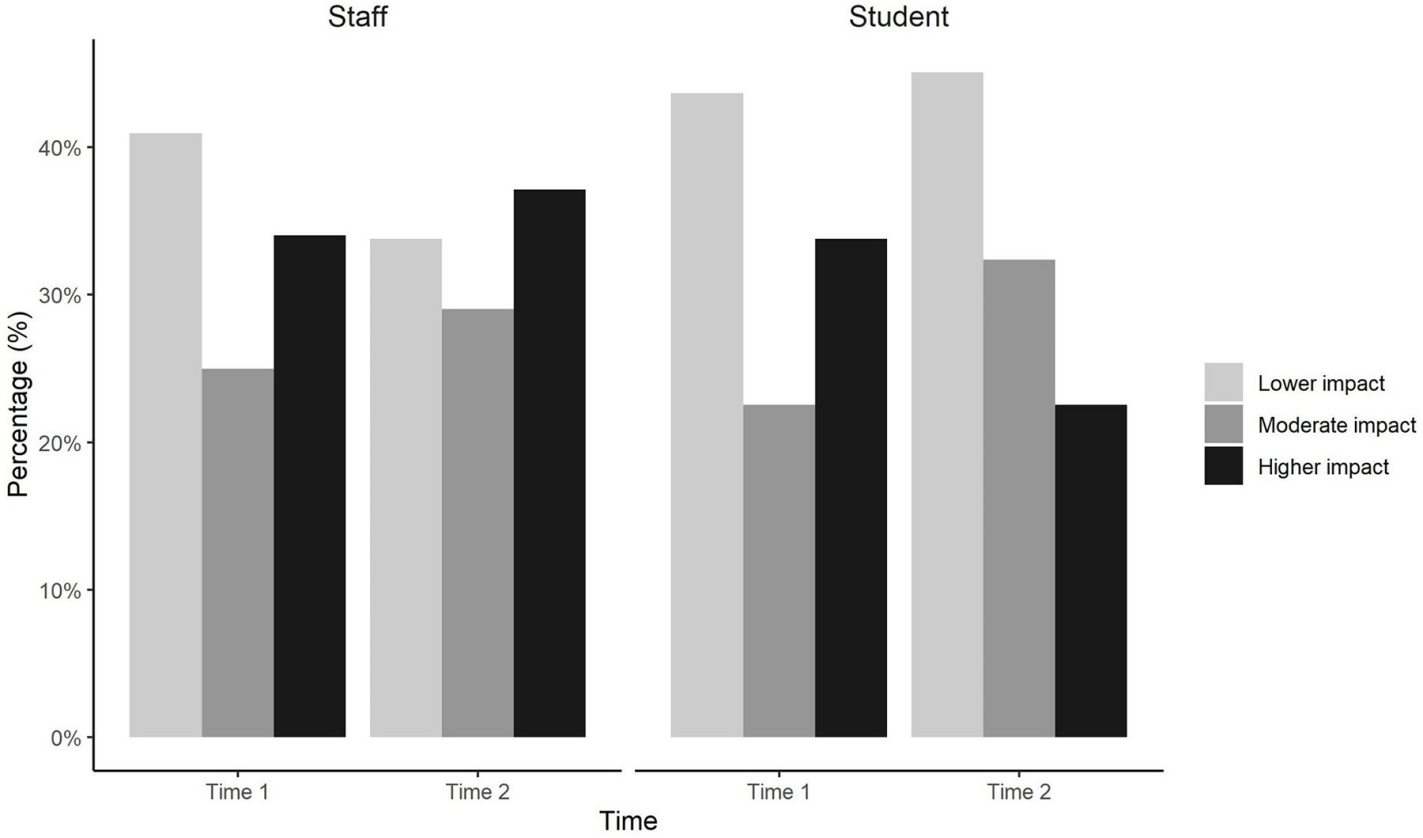
Impact of COVID-19 vaccination on confusion surrounding vaccination in general among staff and students at Time 1 and Time 2.

Impact of COVID-19 vaccination on confusion about vaccination in general is shown in Fig 2. Regarding Time 1 (April-June 2021; participants not yet primary COVID-19 vaccinated), 41.3% of participants (41.0% of staff and 43.7% of students) seemed to perceive less impact, 24.2% (25.0% of staff and 22.5% of students) a moderate impact and 34.4% (34.0% of staff and 33.8% of students) a higher impact. Regarding Time 2 (July-September 2022); participants had not yet received a second booster dose, 35.4% of participants (33.8% of staff and 45.1% of students) seemed to perceive less impact, 28.7% (29.0% of staff and 32.4% of students) moderate impact and 35.8% (37.1% of staff and 22.5% of students) higher impact.

The change of the impact of the COVID-19 vaccination on confusion about vaccination in general is shown in Table 3. There was no change for 55.2% of participants (54.5% of staff and 56.3% of students), an increase in confusion for 25.5% of participants (26.2% of staff (CI 95%: 22.0–30.7) and 19.7% of students (CI 95%: 11.2–30.9)) and a decrease in confusion for 19.4% of participants (19.3% of staff (CI 95%: 15.6–23.4) and 23.9% of students (CI 95%: 14.6–35.5)). The difference between staff and students was insignificant.

**Table 3:** Change of the impact of the COVID-19 vaccination on confusion about vaccination in general among participants included in Time 1 and Time 2 (N = 491).

	All (N = 491)	Staff (N = 420)	Students (N = 71)
Variable	N (%)	N (%)	N (%)
Confusion about vaccination in general			
Decrease	95 (19.4)	81 (19.3)	17 (23.9)
Unchanged	271 (55.2)	229 (54.5)	40 (56.3)
Increase	125 (25.5)	110 (26.2)	14 (19.7)

Abbreviation: N, number

### Factors influencing the impact of COVID-19 vaccination on confusion about vaccination in general at Time 1, Time 2 and its change

Results of univariate and multivariate analyses of factors influencing impact of vaccination against COVID-19 on confusion about vaccination in general at Time 1 among staff members and students are presented in Table 4.

**Table 4:** Results of univariate and multivariate ordinal logistical regressions analyses of factors influencing impact of vaccination against COVID-19 on confusion about vaccination in general at Time 1 among staff members and students (modelling the probability of increasing impact).

	Staff					Students				
	Univariate			Multivariate (n = 419)		Univariate			Multivariate (n = 71)	
Variable	n	OR (95%CI)	P	OR (95%CI)	P	n	OR (95%CI)	P	OR (95%CI)	P
Gender	420		0.64			71		**0.036**		0.28
Female (vs male)		1.09 (0.76–1.57)					2.66 (1.07–6.94)		1.98 (0.58–7.02)	
Age (years)	420	0.99 (0.97–1.01)	0.21			71	0.98 (0.91–1.06)	0.63		
Highest level of education	420		**0.0011**		0.32	71		0.28		
Bachelor’s degree (vs. High school and lower)		0.50 (0.18–1.32)		0.95 (0.30–2.80)			2.73 (0.46–21.73)			
University (vs. High School and lower)		0.28 (0.10–0.71)*		0.68 (0.22–1.97)			0.66 (0.26–1.65)			
Body Mass Index (kg/m^2^)	420	1.00 (0.96–1.04)	0.94			71	0.97 (0.86–1.10)	0.64		
Chronic diseases	420	1.04 (0.78–1.38)	0.79			71	1.20 (0.42–3.46)	0.72		
Careful person	420	0.98 (0.97–0.99)	**0.0026**	0.98 (0.97–1.00)	**0.014**	71	0.98 (0.96–1.01)	0.24		
Careful about health	420	1.00 (0.99–1.01)	0.42			71	0.99 (0.97–1.01)	0.44		
More likely to be sick	420	1.02 (1.01–1.03)	**0.0001**	1.01 (1.00–1.02)	**0.025**	71	1.01 (0.99–1.04)	0.30		
Anxious person	420	1.00 (0.99–1.01)	0.97			71	1.02 (1.00–1.03)	**0.028**	1.01 (0.99–1.03)	0.33
Health literacy	420	1.03 (1.02–1.04)	**<0.0001**	1.02 (1.01–1.04)	**<0.0001**	71	1.05 (1.02–1.09)	**0.0002**	1.04 (1.01–1.08)	**0.015**
Vaccination record	420		**0.0026**		0.053	71		0.51		
No (vs. Yes)		1.25 (0.84–1.86)		1.30 (0.84–2.02)			2.04 (0.40–11.43)			
I don’t know (vs. Yes)		2.56 (1.50–4.43)*		2.06 (1.13–3.79)			1.78 (0.47–6.95)			
Last tetanus booster	420		0.058			71		**0.023**		0.053
I don’t know (vs. I know)		1.41 (0.99–2.01)					2.77 (1.15–6.88)		3.02 (0.99–9.68)	
Adverse event after a vaccination	420		**0.0003**		**0.039**	71		**0.011**		0.074
Event (vs. No event)		1.05 (0.69–1.60)		1.03 (0.61–1.73)			4.21 (1.63–11.34)*		3.60 (1.12–12.31)	
I don’t know (vs. No event)		5.98 (2.44–16.86)*		4.03 (1.36–13.11)*			1.25 (0.15–8.68)		0.63 (0.04–6.97)	
Adverse event after a vaccination (entourage)	420		**0.0028**		0.45	71		0.42		
Event (vs. No event)		1.30 (0.90–1.90)		1.35 (0.85–2.14)			1.74 (0.63–4.97)			
I don’t know (vs. No event)		3.13 (1.61–6.28)*		1.17 (0.51–2.68)			2.36 (0.58–10.01)			
Events that discourage vaccination	420		**0.019**		0.54	71		0.88		
Yes (vs. No)		3.01 (1.20–8.22)		1.40 (0.48–4.34)			1.24 (0.05–33.66)			
Events that encourage vaccination	420		0.19			71		0.38		
No (vs. Yes)		1.33 (0.87–2.05)					0.65 (0.24–1.73)			
Trust granted in priority by the authorities	420		**0.0021**		0.36	71		0.26		
Electoral and macroeconomics interests (vs. Health of the population)		1.77 (1.23–2.55)		1.22 (0.80–1.85)			0.61 (0.25–1.45)			
Trust vaccines producers	420	0.98 (0.98–0.99)	**<0.0001**	1.00 (0.99–1.01)	0.78	71	0.98 (0.96–1.00)	**0.029**	1.00 (0.98–1.03)	0.69
Preferred source information	419		**0.0030**		0.065	71		0.29		
Scientists		0.69 (0.46–1.03)		1.43 (0.87–2.39)			0.36 (0.13–1.01)			
Participant’s entourage		1.38 (0.43–4.57)		1.43 (0.40–5.22)			0.64 (0.02–18.66)			
Health professionals		1.72 (1.04–2.86)*		2.50 (1.28–4.94)			0.54 (0.14–2.06)			
Trust source information	419	0.98 (0.97–0.99)*	**<0.0001**	0.98 (0.97–0.99)	**0.0053**	71	0.97 (0.94–1.00)	**0.018**	1.00 (0.96–1.03)	0.87
Conspiratorial beliefs	420	1.03 (1.02–1.04)	**<0.0001**	1.01 (1.00–1.02)	0.17	71	1.05 (1.02–1.08)	**<0.0001**	1.02 (0.98–1.06)	0.31
Prior SARS-CoV-2 infection (before Time 1)	420		0.46			71		0.98		
Yes (vs. No)		1.19 (0.74–1.92)					0.99 (0.37–2.61)			
Primo-vaccination intention (end of 2020)	420	0.98 (0.98–0.99)	**<0.0001**	0.99 (0.99–1.00)	0.19	71	0.98 (0.97–1.00)	**0.0081**	1.01 (0.99–1.04)	0.35
Primo-vaccination intention (at SARSSURV enrollment)	420	0.97 (0.96–0.98)	**<0.0001**	1.00 (0.98–1.01)	0.80	71	0.96 (0.93–0.98)	**<0.0001**	0.96 (0.92–1.00)	**0.033**

n, number; OR, odds ratio; P, p-value

*, significant p‐value < 0.05

Among staff members, multivariate analysis showed that determinants; careful person, to be sick, health literacy, to have had an adverse event after a past vaccination in general and trust in preferred source of information, were significantly associated with the impact of COVID-19 vaccination on confusion about vaccination in general. People who took better care of themselves were less likely to be more confused about vaccination in general (OR = 0.98 (95% CI: 0.97–1.00)). Participants who were more often sick (OR = 1.01 (1.00–1.02)) with lower health literacy (OR = 1.02 (1.01–1.04)), were more likely to be more confused about vaccination in general. Staff members who didn’t know if they had an adverse event after a past vaccination were more likely to be more confused about vaccination in general compared to participants who reported no prior adverse event (OR = 4.03 (1.36–13.11)). If they showed a higher level of trust in their preferred source of information, there was a lower likelihood that participants would be more confused about vaccination in general (OR = 0.98 (0.97–0.99)).

Multivariate analysis of students revealed that health literacy and primo-vaccination intention at the time of SARSSURV enrolment were significantly associated with confusion about vaccination in general. The lower the level of health literacy, the more likely participants were to be more confused about vaccination in general (OR = 1.04 (1.01–1.08)). The higher the level of primo-vaccination intention of participants at SARSSURV enrolment, the less likely they were to be more confused about vaccination in general (OR = 0.96 (0.92–1.00)).

The results of univariate and multivariate analyses of factors influencing confusion about vaccination in general at Time 2 among staff members and students are shown in Table 5.

**Table 5:** Results of univariate and multivariate ordinal logistical regressions analyses of factors influencing impact of vaccination against COVID-19 on confusion about vaccination in general at Time 2 among staff members and students (modelling the probability of increasing impact).

	Staff					Students				
	Univariate			Multivariate (n = 362)		Univariate			Multivariate (n = 71)	
Variable	n	OR (95%CI)	P	OR (95%CI)	P	n	OR (95%CI)	P	OR (95%CI)	P
Gender	420		**0.021**		0.053	71		0.59		
Female (vs male)		1.53 (1.07–2.21)		1.55 (0.99–2.42)			1.29 (0.51–3.31)			
Age (years)	420	0.99 (0.97–1.00)	0.14			71	0.95 (0.87–1.02)	0.17		
Highest level of education	420		**0.0030**		0.32	71		0.30		
Bachelor’s degree (vs. High school and lower)		0.28 (0.09–0.72)*		0.54 (0.12–2.08)			2.79 (0.38–24.54)			
University (vs. High School and lower)		0.22 (0.08–0.54)*		0.78 (0.18–2.95)			0.64 (0.25–1.59)			
Body Mass Index (kg/m^2^)	420	1.00 (0.96–1.04)	0.96			71	0.88 (0.77–1.00)	0.054		
Chronic diseases	420	1.08 (0.79–1.46)	0.63			71	0.92 (0.30–2.58)	0.88		
Careful person	420	0.98 (0.97–0.99)	**0.0005**	0.98 (0.97–1.00)	**0.0078**	71	0.97 (0.94–1.00)	**0.037**	0.99 (0.96–1.02)	0.61
Careful about health	420	1.00 (0.99–1.01)	0.60			71	0.98 (0.96–0.99)	**0.012**	0.97 (0.95–1.00)	**0.027**
More likely to be sick	420	1.01 (1.00–1.02)	**0.038**	1.01 (1.00–1.02)	0.23	71	1.01 (0.99–1.03)	0.52		
Anxious person	420	1.00 (1.00–1.01)	0.31			71	1.00 (0.99–1.02)	0.69		
Health literacy	420	1.03 (1.02–1.04)	**<0.0001**	1.02 (1.00–1.03)	**0.0058**	71	1.02 (1.00–1.05)	0.070		
Vaccination record	420		**0.0012**		**0.023**	71		0.75		
No (vs. Yes)		1.07 (0.72–1.59)		0.84 (0.52–1.34)			1.81 (0.29–11.45)			
I don’t know (vs. Yes)		2.64 (1.55–4.59)*		2.16 (1.14–4.16)*			1.37 (0.34–5.37)			
Last tetanus booster	420		0.39			71		**0.042**	2.55 (0.98–6.79)	0.055
I don’t know (vs. I know)		1.17 (0.82–1.66)					2.52 (1.03–6.28)			
Adverse event after a vaccination	420		0.68			71		0.30		
Event (vs. No event)		0.96 (0.63–1.46)					1.79 (0.70–4.61)			
I don’t know (vs. No event)		1.40 (0.63–3.20)					0.41 (0.02–3.72)			
Adverse event after a vaccination (entourage)	420		0.17			71		0.20		
Event (vs. No event)		0.90 (0.62–1.30)					0.91 (0.34–2.43)			
I don’t know (vs. No event)		1.68 (0.88–3.26)					3.12 (0.74–14.08)			
Events that discourage vaccination	420		0.70			71		/		
Yes (vs. No)		1.19 (0.50–2.84)					/			
Events that encourage vaccination	420		0.16			71		0.48		
No (vs. Yes)		1.35 (0.89–2.06)					0.70 (0.26–1.91)			
Trust granted in priority by the authorities	420		**<0.0001**		0.17	71		0.55		
Electoral and macroeconomics interests (vs. Health of the population)		2.40 (1.66–3.48)		1.37 (0.87–2.16)			1.31 (0.54–3.17)			
Trust vaccines producers	420	0.98 (0.97–0.99)	**<0.0001**	0.99 (0.98–1.00)	**0.047**	71	1.00 (0.98–1.02)	0.98		
Preferred source information	419		**0.0002**		**0.043**	71		0.089		
Scientists		0.52 (0.34–0.77)*		0.51 (0.30–0.87)*			0.44 (0.16–1.21)			
Participant’s entourage		1.71 (0.55–5.94)		1.99 (0.44–10.77)			3.58 (0.27–88.86)			
Health professionals		1.32 (0.79–2.21)		0.69 (0.32–1.46)			1.55 (0.40–6.13)			
Trust source information	419	0.98 (0.97–0.99)	**<0.0001**	1.00 (0.99–1.02)	0.54	71	0.98 (0.96–1.00)	0.11		
Conspiratorial beliefs	420	1.04 (1.03–1.06)	**<0.0001**	1.02 (1.01–1.04)	**0.0043**	71	1.03 (1.00–1.05)	**0.016**	1.02 (0.99–1.05)	0.25
Prior SARS-CoV-2 infection (before Time 1)	420		0.50			71		0.43		
Yes (vs. No)		0.85 (0.53–1.36)					1.48 (0.56–3.93)			
Prior SARS-CoV-2 infection (before Time 2)	420		0.81			71		0.54		
Yes (vs. No)		0.95 (0.65–1.40)					0.74 (0.29–1.92)			
Primo-vaccination intention (end of 2020)	420	0.99 (0.98–0.99)	**<0.0001**	1.00 (0.99–1.01)	0.88	71	0.99 (0.98–1.01)	0.35		
Primo-vaccination intention (at SARSSURV enrollment)	420	0.97 (0.96–0.98)	**<0.0001**	0.99 (0.97–1.00)	0.13	71	0.98 (0.96–1.00)	**0.038**	0.99 (0.97–1.02)	0.47
Booster intention	363	0.98 (0.97–0.99)	**<0.0001**	0.99 (0.98–1.01)	0.47	59	1.01 (0.98–1.03)	0.53		
Second booster intention	420	0.99 (0.98–0.99)	**<0.0001**	0.99 (0.99–1.00)	0.12	71	1.00 (0.98–1.01)	0.65		
Vaccine status (before Time 2)	420		**0.04**		0.26	71		0.18		
Complete (vs. Incomplete)		0.55 (0.31–0.96)		1.65 (0.68–3.98)			3.11 (0.63–22.77)			
Vaccination symptoms (before Time 2)	416	1.06 (0.98–1.14)	0.14			71	0.90 (0.71–1.13)	0.38		
Neutralizing antibodies (before Time 2)	420	1.00 (1.00–1.00)	0.46			71	1.00 (1.00–1.00)	0.50		

n, number; OR, odds ratio; P, p-value

*, significant p‐value < 0.05

Among staff members, multivariate analysis showed that determinants; careful person, health literacy, to have a record vaccination, trust towards vaccines producers, preferred source of information and conspiratorial beliefs, were significantly associated with the impact of vaccination against COVID-19 on confusion about vaccination in general. The more careful participants were, the less likely they were to be more confused about vaccination in general (OR = 0.98 (0.97–1.00)). The participants with lower health literacy, were more likely to be more confused about vaccination in general (OR = 1.02 (1.00–1.03)). Staff members who did not know if they had a vaccination record were more likely to be more confused about vaccination in general compared to participants who had a vaccination record (OR = 2.16 (1.14–4.16)). The higher trust in vaccines producers, the less likely participants were to be more confused about vaccination in general (OR = 0.99 (0.98–1.00)). Participants who gave scientists as their preferred source of information were less likely to be more confused about vaccination in general (OR = 0.51 (0.30–0.87)). The higher their level of agreement with the anti-vaccination arguments that were circulating during the pandemic, the more likely participants were to be confused about vaccination in general (OR = 1.02 (1.01–1.04)).

Multivariate analysis of students showed that the determinant “careful about health” was significantly associated with the impact of vaccination against COVID-19 on confusion about vaccination in general. The higher their level of care about their health, the less likely participants were to be confused about vaccination in general (OR = 0.97 (0.95–1.00)).

Results of univariate and multivariate analyses of factors influencing the change of impact of vaccination against COVID-19 on confusion about vaccination in general among staff members and students are shown in Tables 6 and 7.

**Table 6:** Results of univariate and multivariate multinomial logistical regressions analyses of factors influencing change of impact of vaccination against COVID-19 on confusion about vaccination in general among staff members.

	Univariate				Multivariate (n = 420)		
	n	Decrease vs Unchanged	Increase vs Unchanged	P	Decrease vs Unchanged	Increase vs Unchanged	P
Variable		OR (95%CI)	OR (95%CI)		OR (95%CI)	OR (95%CI)	
Gender	420			0.17			
Female (vs male)		1.14 (0.68–1.92)	1.58 (0.98–2.57)				
Age (years)	420	0.99 (0.96–1.01)	0.99 (0.96–1.01)	0.44			
Highest level of education	420			0.70			
Bachelor’s degree (vs. High school and lower)		1.11 (0.32–3.81)	0.85 (0.27–2.71)				
University (vs. High School and lower)		0.92 (0.28–3.00)	1.14 (0.38–3.40)				
Body Mass Index (kg/m^2^)	420	1.00 (0.95–1.06)	0.98 (0.93–1.03)	0.72			
Chronic diseases	420	0.98 (0.64–1.50)	0.95 (0.65–1.39)	0.97			
Careful person	420	1.01 (0.99–1.02)	1.00 (0.98–1.01)	0.78			
Careful about health	420	1.01 (0.99–1.02)	1.01 (1.00–1.03)*	**0.044**	1.01 (1.00–1.02)	1.02 (1.00–1.03)*	**0.042**
More likely to be sick	420	1.01 (1.00–1.02)	1.00 (0.99–1.01)	0.085			
Anxious person	420	1.00 (0.99–1.01)	1.00 (1.00–1.01)	0.51			
Health literacy	420	0.99 (0.98–1.00)	0.99 (0.97–1.00)	0.10			
Vaccination record	420			0.82			
No (vs. Yes)		1.08 (0.62–1.89)	0.77 (0.46–1.29)				
I don’t know (vs. Yes)		0.93 (0.43–2.01)	0.95 (0.49–1.84)				
Last tetanus booster	420			0.59			
I don’t know (vs. I know)		1.09 (0.66–1.81)	0.82 (0.52–1.30)				
Adverse event after a vaccination	420			0.13			
Event (vs. No event)		1.35 (0.74–2.45)	1.06 (0.62–1.82)				
I don’t know (vs. No event)		2.14 (0.83–5.52)	0.34 (0.07–1.55)				
Adverse event after a vaccination (entourage)	420			0.083			
Event (vs. No event)		1.97 (1.14–3.39)	1.11 (0.69–1.79)				
I don’t know (vs. No event)		1.42 (0.60–3.34)	0.58 (0.24–1.42)				
Events that discourage vaccination	420			0.063			
Yes (vs. No)		0.86 (0.27–2.73)	0.15 (0.02–1.18)				
Events that encourage vaccination	420			**0.024**			**0.023**
No (vs. Yes)		0.47 (0.26–0.85)*	0.59 (0.34–1.02)		0.47 (0.26–0.84)*	0.58 (0.34–1.01)	
Trust granted in priority by the authorities	420			0.46			
Electoral and macroeconomics interests (vs. Health of the population)		0.85 (0.50–1.43)	1.22 (0.77–1.93)				
Trust vaccines producers	420	1.01 (1.00–1.02)	1.00 (0.99–1.01)	0.34			
Preferred source information	419			0.44			
Scientists		1.58 (0.87–2.86)	0.93 (0.56–1.56)				
Participant’s entourage		1.89 (0.33–10.98)	2.47 (0.63–9.67)				
Health professionals		1.48 (0.73–3.02)	0.77 (0.40–1.49)				
Trust source information	419	1.01 (0.99–1.02)	1.01 (1.00–1.02)	0.30			
Conspiratorial beliefs	420	1.00 (0.99–1.01)	1.00 (0.99–1.01)	0.95			
Prior SARS-CoV-2 infection (before Time 1)	420			0.78			
Yes (vs. No)		0.99 (0.51–1.93)	0.80 (0.43–1.51)				
Prior SARS-CoV-2 infection (before Time 2)	420			0.45			
Yes (vs. No)		0.79 (0.46–1.37)	0.75 (0.46–1.22)				
Primo-vaccination intention (end of 2020)	420	1.00 (0.99–1.01)	1.00 (1.00–1.01)	0.47			
Primo-vaccination intention (at SARSSURV enrollment)	420	1.01 (0.99–1.02)	1.01 (0.99–1.02)	0.50			
Booster intention	363	1.00 (0.99–1.01)	1.00 (0.99–1.01)	0.99			
Second booster intention	420	1.00 (0.99–1.01)	1.00 (0.99–1.00)	0.73			
Vaccine status (before Time 2)	420			0.75			
Complete (vs. Incomplete)		0.75 (0.35–1.60)	0.96 (0.46–1.99)				
Vaccination symptoms (before Time 2)	416	1.04 (0.94–1.16)	1.05 (0.95–1.15)	0.54			
Neutralizing antibodies (before Time 2)	420	1.00 (1.00–1.00)	1.00 (1.00–1.00)	0.61			

n, number; OR, odds ratio; P, p-value

*, significant p‐value < 0.05

**Table 7:** Results of univariate and multivariate multinomial logistical regressions analyses of factors influencing change of impact of vaccination against COVID-19 on confusion about vaccination in general among students.

	Univariate				Multivariate (n = 71)		
	n	Decrease vs Unchanged	Increase vs Unchanged	P	Decrease vs Unchanged	Increase vs Unchanged	P
Variable		OR (95%CI)	OR (95%CI)		OR (95%CI)	OR (95%CI)	
Gender	71			0.081			
Female (vs male)		5.00 (1.00–24.90)	1.20 (0.34–4.24)				
Age (years)	71	1.04 (0.95–1.14)	1.03 (0.93–1.14)	0.66			
Highest level of education	71			0.97			
Bachelor’s degree (vs. High school and lower)		0.78 (0.07–8.52)	1.17 (0.10–13.36)				
University (vs. High School and lower)		1.02 (0.31–3.33)	1.53 (0.43–5.45)				
Body Mass Index (kg/m^2^)	71	1.02 (0.88–1.18)	0.99 (0.84–1.17)	0.96			
Chronic diseases	71	0.90 (0.26–3.09)	0.40 (0.06–2.94)	0.59			
Careful person	71	1.01 (0.97–1.05)	1.00 (0.96–1.03)	0.75			
Careful about health	71	1.03 (1.00–1.06)	1.00 (0.98–1.03)	0.14			
More likely to be sick	71	1.02 (0.99–1.05)	1.01 (0.98–1.04)	0.40			
Anxious person	71	1.02 (1.00–1.04)	1.00 (0.98–1.02)	0.16			
Health literacy	71	1.04 (1.01–1.07)*	1.02 (0.98–1.05)	**0.035**	1.04 (1.01–1.07)*	1.02 (0.99–1.06)	**0.040**
Vaccination record	71			1.00			
No (vs. Yes)		0.76 (0.07–7.98)	0.97 (0.09–10.32)				
I don’t know (vs. Yes)		0.91 (0.16–5.29)	1.16 (0.20–6.88)				
Last tetanus booster	71			0.89			
I don’t know (vs. I know)		0.77 (0.25–2.44)	0.83 (0.24–2.83)				
Adverse event after a vaccination	71			0.43			
Event (vs. No event)		2.47 (0.75–8.16)	0.58 (0.13–2.47)				
I don’t know (vs. No event)		1.79 (0.14–22.70)	1.25 (0.10–15.38)				
Adverse event after a vaccination (entourage)	71			0.49			
Event (vs. No event)		1.50 (0.39–5.75)	0.50 (0.13–1.92)				
I don’t know (vs. No event)		0.39 (0.04–4.28)	0.52 (0.08–3.36)				
Events that discourage vaccination	71			/			
Yes (vs. No)		/	/				
Events that encourage vaccination	71			0.82			
No (vs. Yes)		0.70 (0.19–2.51)	0.73 (0.18–2.88)				
Trust granted in priority by the authorities	71			0.12			
Electoral and macroeconomics interests (vs. Health of the population)		0.29 (0.09–0.96)	0.72 (0.21–2.49)				
Trust vaccines producers	71	0.99 (0.97–1.01)	1.03 (1.00–1.06)*	**0.022**	0.99 (0.97–1.02)	1.03 (1.00–1.06)*	**0.025**
Preferred source information	71			**/**			
Scientists		/	/				
Participant’s entourage		/	/				
Health professionals		/	/				
Trust source information	71	1.00 (0.97–1.02)	1.03 (0.99–1.08)	0.24			
Conspiratorial beliefs	71	1.01 (0.99–1.04)	0.98 (0.94–1.02)	0.18			
Prior SARS-CoV-2 infection (before Time 1)	71			0.75			
Yes (vs. No)		0.81 (0.22–3.03)	1.46 (0.40–5.35)				
Prior SARS-CoV-2 infection (before Time 2)	71			0.73			
Yes (vs. No)		1.16 (0.34–3.97)	1.77 (0.42–7.44)				
Primo-vaccination intention (end of 2020)	71	0.99 (0.98–1.01)	1.02 (0.99–1.04)	0.19			
Primo-vaccination intention (at SARSSURV enrollment)	71	0.99 (0.97–1.01)	1.05 (0.99–1.11)	0.051			
Booster intention	59	1.00 (0.97–1.03)	1.03 (0.98–1.08)	0.40			
Second booster intention	71	1.00 (0.98–1.02)	1.02 (0.99–1.04)	0.35			
Vaccine status (before Time 2)	71			/			
Incomplete (vs. Complete)		/	/				
Vaccination symptoms (before Time 2)	71	0.93 (0.70–1.25)	0.95 (0.70–1.30)	0.88			
Neutralizing antibodies (before Time 2)	71	1.00 (1.00–1.00)	1.00 (1.00–1.00)	0.86			

Abbreviations: n, number; OR, odds ratio; P, p-value

*, significant p‐value < 0.05

Multivariate analysis of staff members showed that those who were careful about their health and recalled events that encourage vaccination were significantly associated with the change in the level of confusion about vaccination in general. The participants who were more careful about their health, were more likely to be more confused about vaccination in general (OR = 1.02 (1.00–1.03)). Participants who had no previous experience of events that encouraged vaccination were less likely to decrease their confusion about vaccination in general (OR = 0.47 (0.26–0.84)) and more likely not to change their mind through the impact of vaccination against COVID-19 on confusion about vaccination in general.

Among students, multivariate analysis showed that health literacy and trust in vaccine producers were significantly associated with the change in the level of confusion about vaccination in general. The participants with a lower level of health literacy, were more likely to be less confused about vaccination in general (OR = 1.04 (1.01–1.07)). The higher their trust in vaccine producers, the more likely participants were to be more confused about vaccination in general (OR = 1.03 (1.00–1.06)).

## Discussion

To the best of our knowledge, this is the first time in Belgium that the factors associated with the impact of vaccination against COVID-19 on the confusion about vaccination in general and the changes over time in scheduling vaccination against COVID-19 have been studied in an academic population, namely staff and students at the University of Liège. Indeed, most articles on the impact of vaccination against COVID-19 on the perception on the vaccination in general are cross-sectional studies [e.g. 45–52] or longitudinal studies mainly conducted during the first waves of the pandemic [e.g. 55, 56] and articles that offer a dynamic approach at different times of the pandemic with a view to understanding such changes are few. The results of the present study will help identify key messages.

### Impact of vaccination against COVID-19 on confusion about vaccination in general

The results of the present study show that of the 491 University of Liège staff members and students who participated in the study, about 25% of the participants demonstrated increased confusion about vaccination in general between prior to primary vaccination against COVID-19 (Time 1) and the second booster dose (Time 2). These results are similar to those of a study conducted in France between 25 April and 9 May 2022, which showed that 30% of participants declared to losing confidence in vaccination programmes in general [53]. In addition, the results of the present study showed that staff members seemed to have more highly increased confusion levels compared to the students (26.2% vs 19.7%, respectively). However, this difference was not significant. This result from the present study showed an opposite trend to that of the study mentioned above which found that younger participants had less confidence than before the COVID-19 vaccination campaign in vaccines in general [53]. However, possible explanations could be that the perception of the usefulness of vaccinating against infectious diseases changes with age [54], information about declining effectiveness in preventing infection and severe illness [42], limited protection against variants [43] requiring several doses in a short period of time. Other explanations are differences in the survey period (April to September 2022 in our study; April and May 2022 in [53]), the study population (university population in our study; adult general population in ☯53]) and the wording of the question to perceive the impact of COVID-19 vaccination on vaccination in general (“What impact has the COVID-19 vaccination campaign had on your confidence in vaccines in general?” in [53]).

In terms of practical implications, the results from the present study showed that the COVID-19 pandemic context reinforced more general vaccine hesitancy. The results also confirmed that *“vaccine hesitancy is complex and context specific*, *varying across time*, *place and vaccines*. *It is influenced by factors such as complacency*, *convenience and confidence*.*”* as mentioned by MacDonald (2015) [9] and reinforces the importance of continuous monitoring of vaccine hesitancy and vaccine decisions for different vaccines after the COVID-19 crisis. Indeed, it is the psychosocial, political, and structural factors which emerge in response to the pandemic which have led to a rise in hesitancy.

### Factors influencing the impact of vaccination against COVID-19 on confusion about vaccination in general

The results have demonstrated the influence of a higher number of individual factors (general self-perception, health literacy, past experiences with vaccination) compared to factors linked to the COVID-19 episode, on confusion towards vaccination in general during the COVID-19 pandemic. Similar results have been produced in other studies which sought to study the determinants of vaccination intention/vaccine attitude during the pandemic. For example, a number of reviews found in the literature highlighted the influence of health literacy [65] and a lack of recent history of flu vaccination [17] on vaccine hesitancy against COVID-19. Other studies have shown that flu vaccination in (previous) flu seasons [46, 48] or the importance of being vaccinated against flu [46] were associated with the intention to have the flu vaccine or a more positive attitude towards receiving the flu vaccine. The results from this study highlight the influence of cognitive aspects (health literacy) and affective aspects on confusion towards vaccination in general. In terms of practical implications, public information and awareness campaigns need to not only focus on understanding vaccines but must also take aspects such as general self-perception into consideration. These results were consistent with the methods proposed by psychology researchers, notably Michie et al. (2020), who recommended a broad spectrum of components to take into account in behavioural monitoring linked to COVID-19 [66]. In addition, it is important to consider factors linked to past vaccination in information campaigns and to offer systematic and differentiated follow-up to people with a negative previous vaccination experience. The influence of individual factors on general vaccination confusion during the COVID-19 pandemic required a more personalised approach to raise awareness of the benefits of vaccination amongst the general public.

With the progress of the pandemic and the COVID-19 vaccination, and in addition to the influence of factors unrelated to the pandemic, a higher number of factors linked to the context (COVID-19 episode) (vaccine producer confidence, preferred source of information, conspiracy beliefs) seem to have impacted confusion towards vaccination in general, particularly among the staff members of the participants. Other studies have found that conspiracy beliefs, scepticism around vaccine production, inadequate or lack of information about the vaccine and a lack of trust in traditional sources of information were predictors of vaccine hesitancy against COVID-19 [21, 36] and that COVID-19 related conspiracy beliefs were associated with general attitudes towards vaccines [67]. These results refer to the impacts caused by the infodemic, also present throughout the COVID-19 pandemic, which have already been mentioned in other studies. Indeed, a systematic examination of reviews showed the effects of infodemics and in particular the impact on reducing patients’ willingness to be vaccinated and increased panic and stress [68], especially since health misinformation on vaccines is common on social media [68]. In terms of practical implications, these results highlight the importance for awareness campaigns of transparent communication based on current knowledge and uncertainties, communicating evidence-based information and adapting messages according to the change of the pandemic while considering the above factors on which it is possible to act (i.e. health literacy) for people to gain a better understanding of the situation. Indeed, increasing health literacy seems to be paramount to fight against the infodemic [68].

### Research prospects

In terms of future research prospects, similar studies need to be carried out on a larger cohort and the general population, in order to develop appropriate public health interventions that inform people more clearly about their confusion around vaccination in general due to the COVID-19 pandemic. Further studies also need to be conducted to measure the real impact of the COVID-19 vaccination on vaccination behaviour in general in a specific context such as ULiège population. In addition, it would be interesting to have a common basic consensus around the concept of vaccine hesitancy (definition, measurement tools, etc.), even if specificities will always have to be taken into account depending on the context. This would facilitate researchers’ understanding and the comparison of results with other studies.

### Strengths of the study

This study was able to go further than previous major studies and to follow changes in the impact of vaccination against COVID-19 on the confusion about vaccination in general at different times of the COVID-19 vaccination schedule, namely before participants had been primary vaccinated (Time 1) and before participants had received the second booster dose (Time 2). The data were also well detailed and documented. Analyses were carried out on a large number of determinants that could have an influence on confusion surrounding vaccination in general, including particular determinants relating to past vaccination as well as vaccination relating to the COVID-19 pandemic. University populations, namely students and staff members, provided data from a closed population, which in turn facilitated action on the factors that influence the impact on vaccination against COVID-19 on the confusion about vaccination in general and its change in a specific population.

### Limits of the study

The results may not be wholly representative of the university population and therefore cannot be applied to the Belgian population at large because (1) participants in the study were a volunteer sample from a highly educated university population with high health literacy, intention to receive a COVID-19 vaccine and generally did so, (2) the cutoff of the impact on vaccination against COVID-19 on the confusion about vaccination in general was calculated based on the tertiles of our sample, and (3) the sample was very small, consisting of only 71 students. There was also a social‐desirability bias that may have influenced the results, although the questionnaires were administered to participants via an online platform in order to minimise bias. Next, and in order to compare data on the participants’ confusion about vaccination in general before and during the COVID-19 pandemic, it would have been interesting to collect data on the participants’ confusion about vaccination in general before the COVID-19 pandemic. Additionally, the question asked on the impact of the COVID-19 pandemic on confusion towards vaccination in general did not make it possible to draw a distinction between participants who seemed less confused and those for whom the pandemic did not seem to have had any impact, not allowing a more detailed description of the impact. A final limitation concerned the questionnaires used to measure the impact of COVID-19 vaccination on vaccine hesitancy in general (confusion about vaccination in general) and the factors influencing it. Although these questionnaires were constructed on the basis of existing literature on vaccine hesitancy and adapted to COVID-19, they did not take into account the measurement models and tools proposed during the COVID-19 pandemic [69–73]. This limitation is explained in particular by the absence of models and questionnaires more specific to the COVID-19 episode in the literature at the time the questionnaires were created.

## Conclusions

In conclusion, the results showed COVID-19 vaccination had a considerable impact on confusion towards vaccination in general among staff members and students in the ULiège community at different times of the pandemic. With the progression of the pandemic and the vaccination, and in addition to the influence of a number of individual factors (general self-perception, health literacy, past experiences with vaccination), a higher number of factors linked to the COVID-19 episode (vaccine producer confidence, preferred source of information, conspiracy beliefs) influenced confusion towards vaccination in general, particularly among staff members. These results have highlighted the importance of a segmented approach based on different population profiles, focusing on the individual’s general self-perception, health literacy, past experiences with vaccination, and adapted to the context and changing situation to a better understanding of vaccination by citizens.

## References

[1] Dubé E, Laberge C, Guay M, Bramadat P, Roy R, Bettinger JA. Vaccine hesitancy: An overview. Human Vaccines & Immunotherapeutics. 2013;9(8):1763‑73. doi: 10.4161/hv.24657 23584253 PMC3906279

[2] Yaqub O, Castle-Clarke S, Sevdalis N, Chataway J. Attitudes to vaccination: A critical review. Social Science & Medicine. 2014;112:1‑11. doi: 10.1016/j.socscimed.2014.04.018 24788111

[3] Larson HJ, Jarrett C, Eckersberger E, Smith DMD, Paterson P. Understanding vaccine hesitancy around vaccines and vaccination from a global perspective: A systematic review of published literature, 2007–2012. Vaccine. 2014;32(19):2150‑9. doi: 10.1016/j.vaccine.2014.01.081 24598724

[4] Peretti-Watel P, Larson HJ, Ward JK, Schulz WS, Verger P. Vaccine hesitancy: clarifying a theoretical framework for an ambiguous notion. PLoS Curr. 2015;7:ecurrents.outbreaks.6844c80ff9f5b273f34c91f71b7fc289. doi: 10.1371/currents.outbreaks.6844c80ff9f5b273f34c91f71b7fc289 25789201 PMC4353679

[5] Bedford H, Attwell K, Danchin M, Marshall H, Corben P, Leask J. Vaccine hesitancy, refusal and access barriers: The need for clarity in terminology. Vaccine. 2018;36(44):6556‑8. doi: 10.1016/j.vaccine.2017.08.004 28830694

[6] Dudley MZ, Privor-Dumm L, Dubé È, MacDonald NE. Words matter: Vaccine hesitancy, vaccine demand, vaccine confidence, herd immunity and mandatory vaccination. Vaccine. 2020;38(4):709‑11. doi: 10.1016/j.vaccine.2019.11.056 31836257

[7] Bussink-Voorend D, Hautvast JLA, Vandeberg L, Visser O, Hulscher MEJL. A systematic literature review to clarify the concept of vaccine hesitancy. Nat Hum Behav. 2022;6(12):1634‑48. doi: 10.1038/s41562-022-01431-6 35995837

[8] WHO. Report of the SAGE Working group on vaccine Hesitancy, 2014 [Internet]. Available from: https://www.who.int/immunization/sage/meetings/2014/october/1_Report_WORKING_GROUP_vaccine_hesitancy_final.pdf

[9] MacDonald NE. Vaccine hesitancy: Definition, scope and determinants. Vaccine. 2015;33(34):4161–4. doi: 10.1016/j.vaccine.2015.04.036 25896383

[10] Larson HJ, Gakidou E, Murray CJL. The Vaccine-Hesitant Moment. New England Journal of Medicine. 2022;387(1):58–65. doi: 10.1056/NEJMra2106441 35767527 PMC9258752

[11] Dubé È, Ward JK, Verger P, MacDonald NE. Vaccine Hesitancy, Acceptance, and Anti-Vaccination: Trends and Future Prospects for Public Health. Annu Rev Public Health. 2021;42(1):175‑91. doi: 10.1146/annurev-publhealth-090419-102240 33798403

[12] Betsch C, Schmid P, Heinemeier D, Korn L, Holtmann C, Böhm R. Beyond confidence: Development of a measure assessing the 5c psychological antecedents of vaccination. PLOS ONE. 2018;13(12). doi: 10.1371/journal.pone.0208601 30532274 PMC6285469

[13] WHO. Ten threats to global health in 2019. 2019 [Internet]. Available from: https://www.who.int/fr/news-room/spotlight/ten-threats-to-global-health-in-2019

[14] WHO. World Health Organization. Director-General’s opening remarks at the media briefing on COVID-19–11 March 2020. 2020 [Internet]. Available from: https://www.who.int/director-general/speeches/detail/who-director-general-s-opening-remarks-at-the-media-briefing-on-covid-19—11-march-2020

[15] Al-Amer R, Maneze D, Everett B, Montayre J, Villarosa AR, Dwekat E, et al. COVID-19 vaccination intention in the first year of the pandemic: A systematic review. Journal of Clinical Nursing. 2022;31:62–86. doi: 10.1111/jocn.15951 34227179 PMC8447353

[16] AlShurman BA, Khan AF, Mac C, Majeed M, Butt ZA. What Demographic, Social, and Contextual Factors Influence the Intention to Use COVID-19 Vaccines: A Scoping Review. International Journal of Environmental Research and Public Health. 2021;18:9342. doi: 10.3390/ijerph18179342 34501932 PMC8431323

[17] Aw J, Seng JJB, Seah SSY, Low LL. COVID-19 Vaccine Hesitancy—A Scoping Review of Literature in High-Income Countries. Vaccines. 2021 Aug 13;9(8):900. doi: 10.3390/vaccines9080900 34452026 PMC8402587

[18] Biswas MR, Alzubaidi MS, Shah U, Abd-Alrazaq AA, Shah Z. A Scoping Review to Find Out Worldwide COVID-19 Vaccine Hesitancy and Its Underlying Determinants. Vaccines. 2021;9:1243. doi: 10.3390/vaccines9111243 34835174 PMC8624792

[19] Wake AD. The Willingness to Receive COVID-19 Vaccine and Its Associated Factors: “Vaccination Refusal Could Prolong the War of This Pandemic”–A Systematic Review. Risk Management and Healthcare Policy. 2021;14:2609–23. doi: 10.2147/RMHP.S311074 34188572 PMC8232962

[20] Kafadar AH, Tekeli GG, Jones KA, Stephan B, Dening T. Determinants for COVID‑19 vaccine hesitancy in the general population: a systematic review of reviews. Journal of Public Health. 2023;31:1829–45. doi: 10.1007/s10389-022-01753-9 36160668 PMC9483252

[21] Romate J, Rajkumar E, Gopi A, Abraham J, Rages J, Lakshmi R, et al. What Contributes to COVID-19 Vaccine Hesitancy? A Systematic Review of the Psychological Factors Associated with COVID-19 Vaccine Hesitancy. Vaccines. 2022;10(11):1777. doi: 10.3390/vaccines10111777 36366286 PMC9698528

[22] Roy DN, Biswas M, Islam E, Azam MS. Potential factors influencing COVID-19 vaccine acceptance and hesitancy: A systematic review. PLoS ONE. 2022;17(3): e0265496. doi: 10.1371/journal.pone.0265496 35320309 PMC8942251

[23] Terry E, Cartledge S, Damery S, Greenfield S. Factors associated with COVID-19 vaccine intentions during the COVID-19 pandemic; a systematic review and meta-analysis of cross-sectional studies. BMC Public Health. 2022;22:1667. doi: 10.1186/s12889-022-14029-4 36056325 PMC9437387

[24] Galanis P, Vraka I, Katsiroumpa A, Siskou O, Konstantakopoulou O, Katsoulas T, et al. First COVID-19 Booster Dose in the General Population: A Systematic Review and Meta-Analysis of Willingness and Its Predictors. Vaccines. 2022;10:1097. doi: 10.3390/vaccines10071097 35891260 PMC9323526

[25] Limbu YB, Huhmann BA. Why Some People Are Hesitant to Receive COVID-19 Boosters: A Systematic Review. Tropical Medicine and Infectious Disease. 2023;8:159. doi: 10.3390/tropicalmed8030159 36977160 PMC10054177

[26] Galanis P, Vraka I, Siskou O, Konstantakopoulou O, Katsiroumpa A, Kaitelidou D. Predictors of COVID-19 vaccination uptake and reasons for decline of vaccination: a systematic review. 2021 [Internet]. Available from: http://medrxiv.org/lookup/. doi: 10.1101/2021.07.28.21261261

[27] Alshahrani NZ, Alsabaani AA, Ridda I, Rashid H, Alzahrani F, Almutairi TH, et al. Uptake of COVID-19 Booster Dose among Saudi Arabian Population. Medicina. 2022;58(7):972. doi: 10.3390/medicina58070972 35888690 PMC9323634

[28] Sprengholz P, Henkel L, Böhm R, Betsch C. Different Interventions for COVID-19 Primary and Booster Vaccination? Effects of Psychological Factors and Health Policies on Vaccine Uptake. Med Decis Making. 2023;43(2):239‑51. doi: 10.1177/0272989X221138111 36404766 PMC9679322

[29] Paridans M, Monseur J, Donneau AF, Gillain N, Husson E, Leclercq D, et al. The Dynamic Relationship between the Intention and Final Decision for the COVID-19 Booster: A Study among Students and Staff at the University of Liège, Belgium. Vaccines. 2022;10(9):1485. 36146562 10.3390/vaccines10091485 PMC9501467

[30] Digregorio M, Van Ngoc P, Delogne S, Meyers E, Deschepper E, Duysburgh E, et al. Vaccine Hesitancy towards the COVID-19 Vaccine in a Random National Sample of Belgian Nursing Home Staff Members. Vaccines. 2022;10(4):598. doi: 10.3390/vaccines10040598 35455347 PMC9028198

[31] Digregorio M, Van Ngoc P, Delogne S, Meyers E, Deschepper E, Dardenne N, et al. Vaccine hesitancy for the COVID-19 vaccine booster dose among nursing home staff fully vaccinated with the primary vaccination course in Belgium. Vaccine: X. 2024;100453. doi: 10.1016/j.jvacx.2024.100453 38361529 PMC10867438

[32] Eichelberger L, Hansen A, Cochran P, Fried R, Hahn M. “In the beginning, I said I wouldn’t get it.”: Hesitant adoption of the COVID-19 vaccine in remote Alaska between November 2020 and 2021. Social Science & Medicine, 2023;116197. doi: 10.1016/j.socscimed.2023.116197 37666096

[33] Hallgren E, Moore R, Purvis RS, Hall S, Willis DE, Reece S, et al. Facilitators to vaccination among hesitant adopters. Human Vaccines & Immunotherapeutics. 2021;17(12):5168–5175. doi: 10.1080/21645515.2021.2010427 34893018 PMC8903968

[34] Moore R, Purvis RS, Willis DE, Li J, Langner J, Gurel-Headley M, et al. “Every Time It Comes Time for Another Shot, It’s a Re-Evaluation”: A Qualitative Study of Intent to Receive COVID-19 Boosters among Parents Who Were Hesitant Adopters of the COVID-19 Vaccine. Vaccines. 2024;12(2), Article 2. doi: 10.3390/vaccines12020171 38400154 PMC10892107

[35] Moore R, Purvis RS, Hallgren E, Willis DE, Hall S, Reece S, et al. Motivations to Vaccinate Among Hesitant Adopters of the COVID-19 Vaccine. Journal of Community Health. 2022;47(2):237–245. doi: 10.1007/s10900-021-01037-5 34687388 PMC8536476

[36] Purvis RS, Hallgren E, Moore RA, Willis DE, Hall S, Gurel-Headley M, McElfish PA. Trusted Sources of COVID-19 Vaccine Information among Hesitant Adopters in the United States. Vaccines. 2021;9(12):1418. doi: 10.3390/vaccines9121418 34960164 PMC8706404

[37] Reece S, CarlLee S, Scott AJ, Willis DE, Rowland B, Larsen K, et al. (2023). Hesitant adopters: COVID-19 vaccine hesitancy among diverse vaccinated adults in the United States. Infectious Medicine. 2023;2(2):89–95. doi: 10.1016/j.imj.2023.03.001 38013742 PMC10038887

[38] Willis DE, Moore R, Selig JP, CarlLee S, Gurel-Headley M, Cornett L, McElfish PA. COVID-19 booster uptake: Are hesitant adopters less likely to get a booster shot than non-hesitant adopters? Behavioral Medicine. 2023. doi: 10.1080/08964289.2023.2249168 PMC11229423 37722699

[39] Willis DE, Selig JP, Andersen JA, Hall S, Hallgren E, Williams M, et al. (2022). Hesitant but vaccinated: Assessing COVID-19 vaccine hesitancy among the recently vaccinated. Journal of Behavioral Medicine. 2022;1–10. doi: 10.1007/s10865-021-00270-6 35032254 PMC8760868

[40] Kashte S, Gulbake A, El-Amin SF III, Gupta A. Covid-19 vaccines: Rapid development, implications, challenges and future prospects. Human Cell. 2021;34(3):711–33. doi: 10.1007/s13577-021-00512-4 33677814 PMC7937046

[41] The Lancet Infectious Diseases. The COVID-19 infodemic. The Lancet Infectious Diseases. 2020;20(8):875. doi: 10.1016/S1473-3099(20)30565-X 32687807 PMC7367666

[42] Feikin DR, Higdon MM, Abu-Raddad LJ, Andrews N, Araos R, Goldberg Y, et al. Duration of effectiveness of vaccines against SARS-COV-2 infection and COVID-19 disease: Results of a systematic review and meta-regression. The Lancet. 2022;399(10328):924–44. doi: 10.1016/S0140-6736(22)00152-0 35202601 PMC8863502

[43] Andrews N, Stowe J, Kirsebom F, Toffa S, Rickeard T, Gallagher E, et al. Covid-19 vaccine effectiveness against the Omicron (b.1.1.529) variant. New England Journal of Medicine. 2022;386(16):1532–46. doi: 10.1056/NEJMoa2119451 35249272 PMC8908811

[44] Agence pour une Vie de Qualité. COVID-19 AVIQ—Les actualités. [Internet]. Available from: https://covid.aviq.be/fr/les-actualites

[45] Antonopoulou V, Goffe L, Meyer CJ, Grimani A, Graham F, Lecouturier J, et al. A comparison of seasonal influenza and novel covid-19 vaccine intentions: A cross-sectional survey of vaccine hesitant adults in England during the 2020 pandemic. Human Vaccines & Immunotherapeutics. 2022;18(5). doi: 10.1080/21645515.2022.2085461 35816683 PMC9621000

[46] Scardina G, Ceccarelli L, Casigliani V, Mazzilli S, Napoletano M, Padovan M, et al. Evaluation of flu vaccination coverage among healthcare workers during a 3 years’ study period and attitude towards influenza and potential COVID-19 vaccination in the context of the pandemic. Vaccines. 2021;9(7):769. doi: 10.3390/vaccines9070769 34358185 PMC8310181

[47] Lennon RP, Block R, Schneider EC, Zephrin L, Shah A. Underserved population acceptance of combination influenza-covid-19 booster vaccines. Vaccine. 2022;40(4):562–7. doi: 10.1016/j.vaccine.2021.11.097 34903376 PMC8650809

[48] Genovese C, Costantino C, Odone A, Trimarchi G, La Fauci V, Mazzitelli F, et al. A knowledge, attitude, and perception study on flu and covid-19 vaccination during the COVID-19 pandemic: Multicentric Italian Survey insights. Vaccines. 2022;10(2):142. doi: 10.3390/vaccines10020142 35214601 PMC8875897

[49] Kwok KO, Li K-K, WEI WI, Tang A, Wong SY, Lee SS. Influenza vaccine uptake, COVID-19 vaccination intention and vaccine hesitancy among nurses: A survey. International Journal of Nursing Studies. 2021;114:103854. doi: 10.1016/j.ijnurstu.2020.103854 33326864 PMC7831770

[50] Sharma B, Racey CS, Booth A, Albert A, Smith LW, Gottschlich A, et al. Characterizing intentions to receive the covid-19 vaccine among the general population in British Columbia based on their future intentions towards the seasonal influenza vaccine. Vaccine: X. 2022;12:100208. doi: 10.1016/j.jvacx.2022.100208 35996447 PMC9387116

[51] Pastorino R, Villani L, Mariani M, Ricciardi W, Graffigna G, Boccia S. Impact of covid-19 pandemic on flu and covid-19 vaccination intentions among university students. Vaccines. 2021;9(2):70. doi: 10.3390/vaccines9020070 33498282 PMC7909275

[52] Mercadante AR, Law AV. Will they, or won’t they? examining patients’ vaccine intention for flu and covid-19 using the health belief model. Research in Social and Administrative Pharmacy. 2021;17(9):1596–605. doi: 10.1016/j.sapharm.2020.12.012 33431259 PMC7833824

[53] Gagneux-Brunon A, Botelho-Nevers E, Verger P, Gauna F, Launay O, Ward JK. Change in self-perceived vaccine confidence in France after the COVID-19 vaccination campaign: A cross-sectional survey in the French general population. Health Policy and Technology. 2024;13(1):100812. doi: 10.1016/j.hlpt.2023.100812

[54] Kessels R, Luyten J, Tubeuf S. Willingness to get vaccinated against covid-19 and attitudes toward vaccination in general. Vaccine. 2021;39(33):4716–22. doi: 10.1016/j.vaccine.2021.05.069 34119349 PMC8149196

[55] Gallant AJ, Nicholls LA, Rasmussen S, Cogan N, Young D, Williams L. Changes in attitudes to vaccination as a result of the COVID-19 pandemic: A longitudinal study of older adults in the UK. PLOS ONE. 2021;16(12). doi: 10.1371/journal.pone.0261844 34941951 PMC8699689

[56] Domnich A, Grassi R, Fallani E, Spurio A, Bruzzone B, Panatto D, et al. Changes in attitudes and beliefs concerning vaccination and influenza vaccines between the first and second COVID-19 pandemic waves: A longitudinal study. Vaccines. 2021;9(9):1016. doi: 10.3390/vaccines9091016 34579253 PMC8470379

[57] Rughiniș C, Vulpe S-N, Flaherty MG, Vasile S. Shades of doubt: Measuring and classifying vaccination confidence in Europe. Vaccine. 2022;40(46):6670–9. doi: 10.1016/j.vaccine.2022.09.039 36216651

[58] Katsiroumpa A, Sourtzi P, Kaitelidou D, Siskou O, Konstantakopoulou O, Galanis P. Predictors of seasonal influenza vaccination willingness among high-risk populations three years after the onset of the COVID-19 pandemic. Vaccines. 2023;11(2):331. doi: 10.3390/vaccines11020331 36851209 PMC9963446

[59] Donneau AF, Guillaume M, Bours V, Dandoy M, Darcis G, Desmecht D, et al. University population-based prospective cohort study of SARS-COV-2 infection and Immunity (SARSSURV-ULiège): A study protocol. BMJ Open. 2022;12(1). doi: 10.1136/bmjopen-2021-055721 35078848 PMC8795924

[60] Von Elm E, Altman DG, Egger M, Pocock SJ, Gøtzsche PC, Vandenbroucke JP. The Strengthening the Reporting of Observational Studies in Epidemiology (STROBE) statement: guidelines for reporting observational studies. The Lancet. 2007;370:1453–57. 10.1016/S0140-6736(07)61602-X 18064739

[61] Larson H, Jarrett C, Schulz W, Chaudhuri M, Zhou Y, Dube E, et al. Measuring vaccine hesitancy: The development of a survey tool. Vaccine. 2015;33:4165–75. doi: 10.1016/j.vaccine.2015.04.037 25896384

[62] Morris NS, MacLean CD, Chew LD, Littenberg B. The Single Item Literacy Screener: Evaluation of a brief instrument to identify limited reading ability. BMC Family Practice. 2006;7(1). doi: 10.1186/1471-2296-7-21 16563164 PMC1435902

[63] Vaccination-info.be. Tétanos. [Internet]. Available from: https://www.vaccination-info.be/maladie/tetanos/

[64] Vaccination-info.be. Grippe. [Internet]. Available from: https://www.vaccination-info.be/maladie/grippe/

[65] Hudson A, Montelpare WJ. Predictors of vaccine hesitancy: Implications for covid-19 public health messaging. International Journal of Environmental Research and Public Health. 2021;18(15):8054. doi: 10.3390/ijerph18158054 34360345 PMC8345367

[66] Michie S, Rubin J, Amlot R. Behavioural science must be at the heart of the public health response to COVID-19. 2020 [Internet]. Available from: https://blogs.bmj.com/bmj/2020/02/28/behavioural-science-must-be-at-the-heart-of-the-public-health-response-to-covid-19

[67] Pivetti M, Di Battista S, Paleari FG, Hakoköngäs E. Conspiracy beliefs and attitudes toward covid-19 vaccinations. Journal of Pacific Rim Psychology. 2021;15:183449092110398. doi: 10.1177/18344909211039893

[68] Borges do Nascimento IJ, Beatriz Pizarro A, Almeida J, Azzopardi-Muscat N, André Gonçalves M, Björklund M, et al. Infodemics and health misinformation: A systematic review of reviews. Bulletin of the World Health Organization. 2022;100(9):544–61. doi: 10.2471/BLT.21.287654 36062247 PMC9421549

[69] Geiger M, Rees F, Lilleholt L, Santana AP, Zettler I, Wilhelm O, et al. Measuring the 7Cs of Vaccination Readiness. European Journal of Psychological Assessment. 2022;38(4):261‑9.

[70] MacDonald NE, Dube E, Comeau JL. Have vaccine hesitancy models oversimplified a complex problem to our detriment? The Adapted Royal Society of Canada vaccine uptake framework. Vaccine. 2022;40(29):3927‑30. doi: 10.1016/j.vaccine.2022.05.052 35637069 PMC9142183

[71] WHO. Data for action: achieving high uptake of COVID-19 vaccines. 2021. Available from: https://iris.who.int/bitstream/handle/10665/340645/WHO-2019-nCoV-vaccination-demand-planning-2021.2-eng.docx?sequence=3

[72] Kotta I, Kalcza-Janosi K, Szabo K, Marschalko EE. Development and Validation of the Multidimensional COVID-19 Vaccine Hesitancy Scale. Human Vaccines & Immunotherapeutics. 2022;18(1):1‑10. doi: 10.1080/21645515.2021.2007708 34919494 PMC8928857

[73] Hammoud H, Albayat SS, Mundodan J, Alateeg S, Adli N, Sabir D, et al. Development and validation of a multi-dimensional COVID-19 vaccine hesitancy questionnaire. Vaccine: X. 2023;14:100286. doi: 10.1016/j.jvacx.2023.100286 36994092 PMC10033149

